# Predictors of overdose response hotline use for mental health and fatal overdose prevention

**DOI:** 10.17269/s41997-024-00981-8

**Published:** 2025-02-18

**Authors:** Will Rioux, Dylan Viste, Taylor Orr, Nathan Rider, S. Monty Ghosh

**Affiliations:** 1 Department of Medicine, Faculty of Medicine & Dentistry, University of Alberta, Edmonton, AB Canada; 2 Department of Medicine, Cumming School of Medicine, University of Calgary, Calgary, AB Canada; 3 Public Health Surveillance and Informatics, Alberta Health Services, Calgary, AB Canada; 4 Department of Internal Medicine, Faculty of Medicine & Dentistry, University of Alberta, Edmonton, AB Canada

**Keywords:** Harm reduction, Mental health supports, Hotline, Opioid crisis, Addiction, Réduction des dommages, Soutiens en santé mentale, Assistance par téléphone, Crise des opioïdes, Dépendance

## Abstract

**Objectives:**

The overdose crisis remains one of the largest public health issues facing North America to date. Formalized virtual spotting services have gained popularity as a harm reduction intervention, proving early effectiveness in reducing overdose mortality. This study determined the characteristics of individuals who recurrently use one such service, Canada’s National Overdose Response Service (NORS).

**Methods:**

In this retrospective study, call logs from NORS were analyzed from service inception. Demographics including age, gender, province, community size, substance used, routes of administration, and adverse events were all collected and imputed into a marginal means and rates model to determine the predictors of recurrent service use.

**Results:**

A total of 7340 unique calls were included within our analysis. Of those, 1167 (15.8%) reported their gender as male, 3744 (51.0%) as female, and 1329 (18.1%) as gender diverse, and 1100 (14.9%) did not report their gender. In terms of age, 46 (0.6%) were individuals under the age of 18 years, 3561 (48.5%) were between 18 and 30, 557 (7.6%) were between 31 and 40, 2505 (34.1%) were between 41 and 50, 525 (7.1%) were age 51 or over, and 146 (2.0%) did not report their age. Men’s rate ratios for recurrent calls were significantly lower than women’s (RR = 0.08, 95% CI = 0.07‒0.09), as were those for respondents aged 31‒40 years as compared with those aged 18‒30 (RR = 0.26, 95% CI = 0.15‒0.45). Between regions, rate ratios for callers from British Columbia (RR = 0.28, 95% CI = 0.17‒2.24) and Atlantic provinces (RR = 0.09; 95% CI = 0.07‒0.12) were significantly lower than those for callers from the province of Ontario. Similarly, rural callers demonstrated lower recurrent service use (RR = 0.08; 95% CI = 0.07‒0.11) than their urban counterparts.

**Conclusion:**

NORS demonstrates higher usage patterns within certain demographic groups, in particular, urban women. The results can therefore be used to target public health messaging toward those who derive the most benefit from the service and to tailor programming to those who are at highest risk to use alone.

## Résumé

**Objectifs:**

La crise des surdoses est encore l’un des plus grands problèmes de santé publique en Amérique du Nord. Les services d’observation virtuelle formalisés gagnent en popularité comme techniques de réduction des méfaits, et selon les données préliminaires, ils s’avèrent efficaces pour réduire les décès par surdose. Nous avons cherché à déterminer les caractéristiques des personnes qui ont actuellement recours à l’un de ces services: le Service national d’intervention en cas de surdose (NORS).

**Méthode:**

Dans le cadre de cette étude rétrospective, nous avons analysé les journaux d’appels du NORS depuis son démarrage. Nous avons recueilli des données démographiques (âge, sexe, province, taille de la communauté, substance consommée, voie d’administration et effets indésirables), que nous avons ensuite entrées dans un modèle de moyennes marginales et de taux marginaux pour déterminer les variables prédictives de l’utilisation répétée du service.

**Résultats:**

Notre analyse a porté sur 7340 appels uniques. Des personnes ayant fait ces appels, 1167 (15,8%) ont dit être des hommes, 3744 (51%) ont dit être des femmes, 1329 (18,1%) ont déclaré une autre identité de genre, et 1100 (14,9%) n’ont pas indiqué leur sexe. Pour ce qui est de l’âge, 46 (0,6%) avaient moins de 18 ans, 3561 (48,5%) avaient de 18 à 30 ans, 557 (7,6%) avaient de 31 à 40 ans, 2505 (34,1%) avaient de 41 à 50 ans, 525 (7,1%) avaient 51 ans et plus, et 146 (2%) n’ont pas indiqué leur âge. Selon l’analyse des appels répétés, les rapports de taux des hommes étaient sensiblement moins élevés que ceux des femmes (RT = 0,08, IC de 95% = 0,07‒0,09); c’était aussi le cas pour les répondants de 31 à 40 ans comparativement à ceux de 18 à 30 ans (RT = 0,26, IC de 95% = 0,15‒0,45). Selon l’analyse par région, les rapports de taux pour les appels de la Colombie-Britannique (RT = 0,28, IC de 95% = 0,17‒2,24) et des provinces de l’Atlantique (RT = 0,09; IC de 95% = 0,07‒0,12) étaient sensiblement moins élevés que ceux pour les appels de l’Ontario. De même, l’utilisation répétée du service était plus faible chez les personnes vivant en zone rurale (RT = 0,08; IC de 95% = 0,07‒0,11) que chez celles vivant en zone urbaine.

**Conclusion:**

Certains groupes démographiques, en particulier les femmes vivant en zone urbaine, ont plus tendance à utiliser le NORS. Nos résultats peuvent donc servir à adresser les messages de santé publique aux groupes qui tirent le meilleur profit de ce service, et à adapter les programmes en fonction de ceux qui courent le plus grand risque de consommer seuls.

## Introduction

The overdose epidemic continues to claim the lives of tens of thousands of individuals across North America and is complicated by an ever-changing drug supply and diverse substance use contexts (Belzak & Halverson, 2018; Imtiaz et al., 2020). Diverse public health interventions and programs have been developed in response to this crisis, including Canada’s four-pillar strategy of prevention, treatment, enforcement, and harm reduction components (Health Canada, 2016). The latter comprises a variety of interventions, including naloxone distribution, supervised consumption sites/overdose prevention sites, needle exchanges, and drug-checking services. More recently, overdose response technologies (also referred to in the literature as virtual overdose monitoring services, overdose response applications and hotlines (ORAH), and mobile overdose response services) have gained popularity as additional harm reduction measures to reach those who cannot or choose not to access other harm reduction measures (Loverock et al., 2023; Viste et al., 2023). Despite emerging evidence, examining these services’ acceptability, uptake, and effectiveness to inform public policy remains critical (Sekhon et al., 2017).

Virtual spotting occurs when individuals reach out to contacts to supervise them during a substance use session to ensure that emergency services are dispatched should they become unresponsive (Perri et al., 2021, 2022). Born out of these grassroots efforts, these services became formalized as overdose response hotlines, allowing anyone with a phone and a cellular connection to access harm reduction support services and overdose monitoring (Loverock et al., 2023; Tay Wee Teck et al., 2023). Recent evidence demonstrates that these services have enabled effective overdose responses (Rioux et al., 2023a, 2023b; Viste et al., 2023), supported mental health of PWUS (people who use substances), and connected PWUS to resources and community, among others (Rider et al., 2023; Viste et al., 2024).

Despite these early successes, uptake remains limited in spite of the potential accessibility of overdose response hotlines (Mocanu et al., 2024; Rioux et al., 2023a, 2023b). We conducted a retrospective observational study of one such service, Canada’s National Overdose Response Service (NORS), to determine the characteristics of individuals with high versus those with low service utilization over time. To our knowledge, this is the first study to examine individual characteristics regarding virtual harm reduction service utilization, the results of which can be used to understand populations who may derive the most benefit from these services and to whom public health messaging should be targeted, and to identify demographic groups for whom adaptations should be tailored to promote recurrent service use and connection to harm reduction interventions.

## Methods

### Setting

The NORS is a multimodal harm reduction service available across Canada that provides virtual supervised consumption services through text and hotline. As the texting service has been a recent addition, the results of this study’s analysis focus only on phone call log data collected between December 15, 2020, and August 31, 2023. Service users are connected to an operator, most often a person with lived or living experience of substance use, who records basic de-identified information described in detail below. During a call, operators note the caller’s full physical address and cell phone/mobile phone information should an emergency response need to be activated or the client needs to be called back. This information is later destroyed. Furthermore, other demographic data, including gender and Indigeneity, began to be collected in July 2022 and were retrospectively applied to callers using unique identifiers.

### Variables and groupings

Variables were collected based on similar data collection strategies employed by Health Canada in their monitoring of physical supervised consumption spaces (Health Canada, 2021). These variables include gender (women, men, and gender diverse), age (18–30, 31–40, 41–50, 51–60, and 60 +), Indigenous identity (yes/no), province, community type (urban [> 100,000 population], medium [10,000–100,000 population], rural [< 10,000 population]), time of call, date of call, call type (supervised consumption, mental health, peer support, and resource referral), the substance used (opioids, cocaine, methamphetamines, depressants, cannabis, polysubstance use, or other), route of use (injection, insufflation, inhalation, oral, poly route, or other), and adverse events (including overdose or acute mental health crisis requiring emergency medical service dispatch). Notably, gender and Indigenous identity were only collected voluntarily and thus were optionally disclosed. Variable groupings were performed to preserve the privacy of individuals when individual data points were less than 10 in a given group category. Groupings include geographic location (Prairie provinces [Alberta (AB), Saskatchewan (SK), Manitoba (MB)], Atlantic provinces [Nova Scotia (NS), New Brunswick (NB), Newfoundland and Labrador (NL), Prince Edward Island (PE)], Northern provinces and territories [Nunavut (NU), Northwest Territories (NT), Yukon Territory (YT)]) and time of calls (early morning (0:00–5:59), morning (6:00–11:59), afternoon (12:00–17:59), and evening (18:00–23:59)). Due to the population size with age identifiers under 18, we elected to consider age entries below the age of 16 as errors and list these clients as missing their age.

### Statistical analysis

To analyze demographic characteristics that predict the utilization of the NORS service, we used a form of survival analysis for recurrent events called the marginal means and rates model (Amorim & Cai, 2015). A survival analysis predicts the time until an event occurs, and in our study, the event of interest is the time between each phone call an individual makes or until they are censored out at the end of the study’s time period. A traditional survival analysis models only up until the first event for an individual and then the individual is censored out of the analysis, ignoring subsequent events (Clark et al., 2003). Thus, the marginal means and rates model accounts for multiple events with each individual by modeling for the rate of events (Amorim & Cai, 2015). Model covariates predict the likelihood of an individual within a particular group making return phone calls to NORS as compared with individuals within a reference group. High service utilization for our study is defined as individuals within a covariate group having a higher rate ratio in comparison with a reference group, whereas low service utilization is defined as a lower rate ratio versus the reference group. The model’s reference groups were chosen based on the covariate characteristics with the highest call frequencies using the total number of calls in the entire dataset (Viste et al., 2023). Thus, we would expect the model outcomes to primarily display covariate groups with significantly lower service utilization as compared with the reference group.

**Fig. 1:**
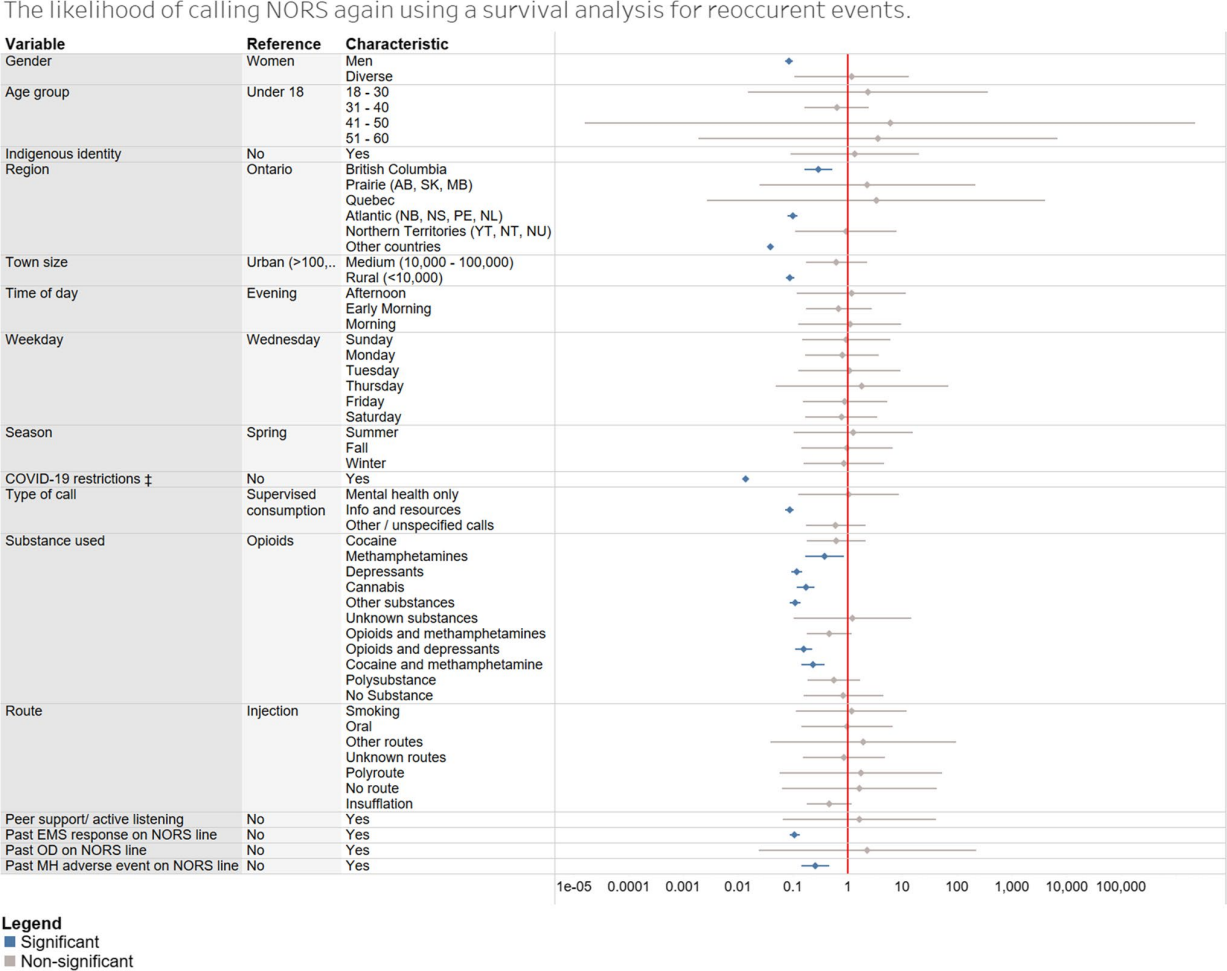
The likelihood of recurrent calls by unique users to the National Overdose Response Service

Call records that could not be determined to be unique individuals were removed due to the lack of demographic data provided (*n* = 440; 5.6% of the total dataset). Additionally, 151 records (2.0%) were missing their age, 1031 records (13.9%) were missing their gender identity, 468 records (6.3%) were missing their town size, and 258 records (3.4%) were missing their region. To address missing data within our demographic indicators (age, gender, town size, and region), multivariate imputation by chained equations (MICE) was used (Azur et al., 2011). The dataset was imputed 30 times over 20 iterations to create 30 imputed datasets. The marginal means and rates model was run on all 30 imputed datasets, and their model coefficients were then averaged to create a final model. Rate ratios and 95% confidence intervals predicting an individual’s likelihood of making a return phone call were calculated using the final model’s averaged coefficients and standard errors. Data cleaning was performed using SAS Enterprise Guide 8.3. Imputation and survival analysis modelling was conducted using R version 4.3.0 with the imputational packages “MICE” and “survival” (Azur et al., 2011; Buuren & Groothuis-Oudshoorn, 2010; Therneau et al., 2024). The forest plot of rate ratios for Fig. 1 was created using Tableau 2023.3. Ethics approval was obtained from the University of Calgary (REB21-1966). This study used the STROBE guidelines to assist in the reporting of the results (Cuschieri, 2019).

## Results

During the study period, the NORS received 7824 calls. Of these records, 484 (6.2%) were removed as they lacked sufficient data to be confirmed as unique callers^1^, leaving a total of 7340. Caller sample characteristics are described using the categorical variables in Table 1. Of note, men were a minority of the sample, as were callers from the Atlantic Region and the Northern Territories. A similar number of the total unique callers were men and women (approximately 13%), but women contributed to a much greater frequency of total calls (51.0%) than men (15.8%). Similarly, NORS had many one-time callers (62.8%) but they only contributed to 5.0% of total calls, whereas nine callers have phoned NORS over 100 times (1.5%) and contributed nearly three quarters of all calls (74.4%).

**Table 1:** Characteristics of total calls from identified unique callers of the NORS hotline and characteristics of unique callers

Characteristic	Total sum of calls (%)	Total count of unique callers (%)
**Total**	**7340**	**582**
Gender		
Men	1167 (15.8)	75 (12.8)
Women	3744 (51.0)	80 (13.7)
Gender diverse	1329 (18.1)	6 (1.0)
Unknown gender	1100 (14.9)	421 (72.3)
Age		
< 18	46 (0.6)	11 (1.9)
18–30	3561 (48.5)	223 (38.3)
31–40	557 (7.6)	133 (22.8)
41–50	2505 (34.1)	60 (10.3)
51–60	470 (6.4)	32 (5.5)
> 60	55 (0.7)	11 (1.9)
Unknown age	146 (2.0)	125 (21.4)
Indigenous identity	592 (8.1)	13 (2.2)
Province or region		
British Columbia	234 (3.2)	60 (10.3)
Prairie (AB, SK, MB)	1513 (20.6)	89 (15.2)
Ontario	4553 (62.0)	327 (56.1)
Quebec	693 (9.4)	43 (7.4)
Atlantic (NB, NS, PE, NL)	44 (0.6)	32 (5.5)
Northern Territories (YT, NT, NU)	21 (0.3)	8 (1.4)
Other countries	24 (0.32)	21 (3.6)
Unknown region	258 (3.5)	91 (15.6)
Size of community		
Urban (>100,000)	6574 (89.5)	431 (74.0)
Medium (10,000–100,000)	256 (3.5)	56 (9.6)
Rural (<10,000)	42 (0.6)	26 (4.5)
Unknown community	468 (6.4)	160 (27.4)
Time of call		
00:00 to 05:59	659 (9.0)	153 (26.2)
06:00–11:59	1671 (22.7)	186 (31.9)
12:00–17:59	2281 (31.0)	239 (41.0)
18:00–23:59	2729 (37.1)	265 (45.5)
Day of week		
Sunday	882 (12.0)	119 (20.4)
Monday	1070 (14.5)	153 (26.2)
Tuesday	1044 (14.2)	131 (22.5)
Wednesday	1120 (15.2)	155 (26.6)
Thursday	1095 (14.9)	116 (19.9)
Friday	1067 (14.5)	141 (24.2)
Saturday	1062 (14.4)	150 (25.7)
Season		
Spring	2296 (31.2)	184 (31.6)
Summer	2144 (29.2)	230 (39.5)
Fall	1324 (18.0)	146 (25.0)
Winter	1576 (21.4)	161 (27.6)
COVID-19 restrictions‡	3700 (50.4)	265 (45.5)
Type of call		
Supervised consumption	4700 (64.0)	407 (69.9)
Mental health or peer support only	2048 (27.9)	228 (39.1)
Info and resources	187 (2.5)	221 (37.9)
Other / unspecified calls	405 (5.5)	57 (9.8)
Mental health and supervised consumption	1043 (14.2)	114 (19.5)
Type of substances used		
Opioids	3525 (48.0)	252 (43.2)
Cocaine	715 (9.7)	135 (23.1)
Methamphetamines	329 (4.5)	68 (11.6)
Depressants*	36 (0.4)	26 (4.5)
Cannabis	30 (0.4)	12 (2.1)
Other substances	15 (0.2)	14 (2.4)
Unknown substances	176 (2.4)	39 (6.7)
Opioids and methamphetamines	54 (0.7)	22 (3.8)
Opioids and depressants*	12 (0.2)	11 (1.9)
Cocaine and methamphetamines	8 (0.1)	5 (0.8)
Polysubstance	188 (2.6)	72 (12.3)
Route of substance used		
Injection	2526 (34.4)	209 (35.9)
Smoking	1577 (21.4)	134 (23.0)
Insufflation / snorting	195 (2.7)	81 (13.9)
Oral	301 (4.1)	57 (9.8)
Other routes	29 (0.4)	12 (2.1)
Unknown routes	407 (5.5)	78 (13.4)
Polyroute	96 (1.3)	28 (4.8)
Peer support / active listening	2646 (36.0)	189 (32.4)
Call outcomes		
All adverse events	119 (1.6)	54 (9.3)
Overdose events	93 (1.3)	41 (7.0)
EMS dispatched	72 (1.0)	26 (4.5)
False alarm EMS dispatches	4 (0.05)	3 (0.5)
Designated emergency contact lay responder	15 (0.2)	6 (1.0)
EMS and designated contact dispatched	4 (0.05)	2 (0.3)
Response unknown	2 (0.02)	2 (0.3)
Mental health adverse events	30 (0.4)	19 (3.3)
Mental health adverse events that also qualified as overdose events	4 (0.05)	2 (0.3)
EMS dispatched	9 (0.1)	6 (1.0)
Designated emergency contact lay responder	3 (0.04)	1 (0.2)
Verbally resolved by staff	15 (0.2)	9 (1.5)
Transferred to another crisis line	3 (0.04)	2 (0.3)
Number of calls		
1 call	368 (5.0)	366 (62.8)
2–10 calls	465 (6.3)	149 (25.6)
11–49 calls	705 (9.6)	30 (5.2)
50–99 calls	318 (4.3)	11 (1.9)
100+ calls	5464 (74.4)	9 (1.5)
Unknown callers	20 (0.3)	19 (3.3)

*AB* Alberta, *SK* Saskatchewan, *MB* Manitoba, *NB* New Brunswick, *NS* Nova Scotia, *PE* Prince Edward Island, *NL* Newfoundland and Labrador, *YT* Yukon Territory, *NT* Northwest Territories, *NU* Nunavut

‡The duration of the Canadian COVID-19 restrictions was defined as between March 1, 2020, and March 1, 2022

*Depressants include depressants such as benzodiazepines and alcohol

Over the operational period (December 15, 2020–August 31, 2023), 93 calls were classified by service operators as overdose events. Of those, 72 required EMS dispatch and naloxone administration, 15 involved notifying trusted community members to respond, and 4 required dispatch of both EMS and community responders; in two instances, the respondent type was not recorded. Sixty-six of these events occurred in the province of Ontario, 9 in British Columbia, 6 in Alberta, 3 in Manitoba, and 1 each in Quebec, Nova Scotia, Prince Edward Island, and Yukon, Five overdose events either occurred in unknown locations or in countries outside of Canada. In terms of substances used, 77 reported the use of opioids (mostly fentanyl), 12 methamphetamines, 6 crack/cocaine, 2 depressants (e.g., benzodiazepines) and 1 alcohol, and in 9 cases the substance used was unknown. In 14 cases, multiple substances were used. Substances were injected in 37 cases of overdose; 32 reported smoking their substances, 3 reported snorting/insufflation of substances, and 6 involved both injecting and smoking; in 15 cases the routes were not disclosed/unknown.

With respect to the utilization by unique users of the NORS, rate ratios for recurrent calls were significantly lower among men than among women (RR = 0.08, 95% CI = 0.07–0.09). Compared to callers aged 18–30, those aged 31–40 had a significantly lower rate ratio for calling NORS (RR = 0.26, 95% CI = 0.15–0.45). Between regions, rate ratios for callers from British Columbia (RR = 0.28, 95% CI = 0.17–2.24), Atlantic provinces (RR = 0.09; 95% CI = 0.07–0.12), and other countries (RR = 0.03; 95% CI = 0.03–0.04) were significantly lower than those for Ontario callers. Individuals from rural locations (RR = 0.08; 95% CI = 0.07–0.11) had significantly lower rate ratios than individuals from urban locations. Also, individuals calling after COVID-19 restrictions (RR = 0.013; 95% CI = 0.013–0.014) had significantly lower rate ratios than individuals calling during COVID-19 restrictions. Callers calling for information and resources (RR = 0.08; 95% CI = 0.07–0.1) had significantly lower rate ratios than callers calling for supervised consumption services. Regarding substance use, individuals using methamphetamines (RR = 0.37; 95% CI = 0.17–0.85), depressants (RR = 0.11; 95% CI 0.09–0.14), cannabis (RR = 0.17; 95% CI = 0.12–0.25), other substances (RR = 0.10; 95% CI = 0.08–0.14), opioids and depressants (RR = 0.15; 95% CI = 0.11–0.22), or cocaine and methamphetamines combined (RR = 0.23; 95% CI = 0.14–0.38) had significantly lower rate ratios than callers consuming opioids. Finally, individuals who experienced a mental health adverse event (RR = 0.25; 95% CI = 0.14–0.45) or EMS response (RR = 0.11; 95% CI = 0.08–0.13) while on the NORS line also had significantly lower rate ratios for calling NORS. Additional rate ratios and confidence intervals for the likelihood of a unique NORS caller being a high-frequency caller based on their characteristics can be found in the Appendix.

## Discussion

In this study of call logs recorded between December 15, 2020, and April 30, 2023, under the National Overdose Response Service, we found that men were significantly less likely to continue service use after an initial call than women. Callers from the 31–40-year-old age group were also less likely to have recurrent calls in comparison to those in the 18–30-year age group; similarly, callers from British Columbia and the Atlantic provinces were much less likely to continue using the NORS after their initial call. Individuals who experienced an overdose or adverse mental health event were also significantly less likely to return to service use compared to callers who did not experience these events. Moreover, in comparison to opioids, nearly all other types of substances used on the line had lower rates of returns to service use. Finally, callers were also less likely to continue to call back during the COVID-19 period as compared with other periods.

### Gender

The finding that men were less likely to use the service again may be attributed to various causes, including higher use of in-person harm reduction facilities (Nassau et al., 2022; Public Health Agency of Canada, 2023) or higher risk use patterns (i.e., using alone), which has been cited in the literature as a contributing factor to the disproportionate overdose mortality seen within this population (Bardwell et al., 2019). Moreover, the higher proportion of women repeatedly engaging with the NORS line may also be attributed to a variety of factors, including but not limited to physical sites as gendered spaces, higher rates of exposure to physical and sexual violence in these spaces, greater substance use stigma among this population and potential complications from childcare (including stigma and loss of child care privileges), and unmet need (Boyd et al., 2018; Perri et al., 2022; Rosen et al., 2022; Xavier et al., 2021). Similarly, mental health hotlines show increased uptake among women (Matthews et al., 2023), and as a result, a similar relationship may be present for NORS. Unfortunately, men are also disproportionately represented within populations of substance users and at greater risk of substance use mortality (Health Canada, 2021). It has been hypothesized that due to previous associations with risk-taking behaviours in men, it is likely that these similar relationships would exist in substance use contexts (Butelman et al., 2023) and may contribute to decreased uptake of harm reduction*.* While discrepancies in service use seen within this study may also be partially attributable to the availability and willingness of males to engage with physical harm reduction facilities, future work should aim to both target men who use drugs alone (Bardwell et al., 2019) and elucidate the underlying reasoning for lower recurrent service uptake within this demographic group. However, it is essential to note that these demographic data are quite limited within our dataset due to the relative recency and the optional nature of their collection.

### Age

Those participants between the ages of 31 and 40 were associated with significantly lower usage than those aged 18–30. While trends coincided with potentially higher technological literacy seen with youth (Paige et al., 2018), this pattern did not continue in older age groups. Limitations within our sample size for this population may partially explain our results; however, future research should consider elucidating rationales behind this usage pattern as this age group is significantly overrepresented within Canadian overdose mortality data (Health Canada, 2021). Furthermore, no similar trend exists among their brick-and-mortar supervised consumption site counterparts, wherein the most significant percentage (34%) of users are between the ages of 30 and 39 (Public Health Agency of Canada, 2023).

### Location

Service utilization varied widely by geographic region, with Atlantic regions, British Columbia, and rural regions demonstrating decreased recurrent usage in comparison with other areas. While substance use patterns and behaviours vary between regions, this relationship may depend in part on whether there is access to harm reduction services such as supervised consumption sites. For example, British Columbia offers similar overdose response services, including the Lifeguard app and the Brave app (Powered by evan | A Digital Health Company, n.d.; The Brave App, n.d.). Increased presence and dissemination of public health information regarding these services would allow PWUS additional accessible resources while partially explaining our results. In contrast, a similar service exists in the province of Alberta (the Digital Overdose Response Service); however, this province does not exhibit the same relationship (DORS App, n.d.). Finally, no such additional services exist within Atlantic provinces.

Our results also demonstrate that rural locations exhibited decreased returns to service usage. This is of particular interest as rural locations often lack access to harm reduction services, which is thought to have contributed, in part, to increased rates of overdose mortality in these regions (Hu et al., 2022). Previous studies have indicated that rural communities often significantly stigmatize substance use, and due to the fear of confidentiality breaches and fears of being “outed” in their community, PWUS are less likely to use harm reduction resources (DeBeck et al., 2012; Mitra et al., 2019). While previously hypothesized that the lack of harm reduction centres and communities may also hinder the uptake and knowledge of virtual service modalities available, one study of the NORS found that a significant majority of those accessing the service did not have access to such services in their community (Mocanu et al., 2024). Continued efforts to increase awareness of these services in rural communities, such as naloxone kit messaging (Safi et al., 2023), should be combined with examining low recurrence of service usage.

### Type of call

Regarding the types of calls, information and resource-based calls were also associated with a decreased return to service usage, in part attributed to individuals calling on behalf of others looking for information on the service or PWUS looking for basic information on harm reduction as has been seen in recent publications (Rider et al., 2023; Viste et al., 2023). Such individuals might have less need to call back.

### Substances used and route of administration

Depressants such as benzodiazepines and alcohol and substances such as cannabis are inherently less risky in terms of mortality risk than substances such as opioids and stimulants (Public Health Agency of Canada, 2022). As such, it is expected that individuals using those substances may utilize the line less frequently.

### Experiencing an adverse mental health event or EMS callouts

Experiencing an event that required EMS dispatch while utilizing the service and adverse mental health events was associated with a lower rate of service use. It can be hypothesized that potentially negative or stigmatizing reactions faced by individuals interacting with the healthcare system, as seen in other studies (Paquette et al., 2018), may dissuade individuals from accessing this and other overdose response hotlines, fearing similar experiences during adverse events on the line. Future studies should be undertaken to clarify this association and provide insight towards reducing these barriers to service access. Overall, the dissemination of knowledge and building of trust in these communities are not well described in the current literature. Future investigations should evaluate the low uptake within these specific populations and determine strategies to target those without access to harm reduction.

### Limitations

A few limitations should be considered when interpreting the results of this study. To begin, due to the small sample size of the population studied, it remains difficult to accurately interpret service usage characteristics, particularly within various communities that demonstrate low service uptake. Second, as mentioned above, multiple overlapping service offerings are present within individual provinces, preventing an accurate description of service uptake within these regions. Additionally, our results are primarily derived from call logs, which are created from self-reported information and may be subject to response bias, particularly due to callers’ privacy concerns and the current criminalization of substance use. These call logs are also manually entered by service operators and, as a result, may be subject to recall biases or other clerical errors. In addition, gender and Indigenous identity were only recorded optionally, which may contribute to response and nonresponse biases. The use of MICE also results in predicted synthetic client data which will result in increased variability for the imputed demographic variables; however, MICE allows records with some missing data to remain in the study, rather than removing their record, and retains important data for other variables of interest. Furthermore, many of the missing demographic data were from clients with multiple calls and a fraction of call logs did not capture their demographic data.

## Conclusion

The findings of this study indicate that some key populations utilize overdose response hotlines more than others, particularly women from urban communities and those using opioids. This study suggests that overdose response hotlines are a valuable option for those demographics using the service at a high rate. Therefore, the findings of this study can be used to target public health messaging towards population groups who may derive the most benefit from these services as well as potential groups for whom adaptations are needed to encourage virtual harm reduction service use or to not use alone*.* Specifically, future work should aim to increase uptake and recurrent use of overdose response hotlines in rural demographics and others who represent a larger burden of mortality from the overdose epidemic and may otherwise lack access to harm reduction.

## Contributions to knowledge

What does this study add to existing knowledge?

- This is the first article to describe the recurrent use patterns of overdose response hotlines and adds to the to-date scarce literature regarding the recurrent use of harm reduction services.
- This article supports previous qualitative literature demonstrating the potential for virtual spotting services like NORS, including usage among women; however, it also demonstrates low recurrent use among rural users who have historically lacked access to harm reduction services in Canada.
- These findings present key avenues for future research and public health decision-making to address these remaining inequities.

What are the key implications for public interventions, practice, or policy?

- Public health interventions should target men, rural populations, and the 31–40-year age group, as these groups are less likely to engage with overdose response services despite high overdose mortality rates. Gender-specific strategies for men and continued support for women’s engagement are crucial.
- Rural communities need outreach to raise awareness and reduce stigma around virtual harm reduction services. Addressing adverse mental health and EMS-related stigma is essential to improving service retention.
- Tailored substance-specific messaging, particularly for non-opioid users, and improved data collection for marginalized groups can enhance public health strategies in harm reduction efforts.

## Data Availability

The datasets generated and/or analyzed during the current study are not publicly available due to privacy concerns around NORS clients’ illicit substance use but are available from the corresponding author upon reasonable request.

## Appendix

Rate ratios and 95% confidence intervals for the likelihood of a unique NORS caller being a high-frequency caller based on their characteristics

**Table:**

Characteristic	Rate ratios (95% CI)
Gender (Ref: Women)	
Men	0.08 (0.07 – 0.09)***
Diverse	1.18 (0.10 – 13.2)
Age group (Ref: 18–30)	
Under 18	0.42 (0.17 – 1.07)
31–40	0.26 (0.15 – 0.45)*
41–50	2.51 (0.01 – 412.0)
51–60	1.52 (0.07 – 31.4)
60 +	0.48 (0.18 – 1.32)
Indigenous identity (Ref: Non-Indigenous)	
Indigenous	1.34 (0.09 – 20.2)
Region (Ref: Ontario)	
British Columbia	0.28 (0.16 – 0.52)*
Prairie (AB, SK, MB)	2.28 (0.17 – 2.69)
Quebec	3.30 (0.12 – 9.43)
Atlantic Canada (NB, NS, PE, NL)	0.09 (0.08 – 0.12)*
Northern Territories (YT, NT, NU)	0.93 (0.11 – 7.82)
Other countries	0.03 (0.03 – 0.04)***
Community size (Ref: Urban (> 100,000))	
Medium (10,000‒100,000)	0.61 (0.17 – 2.25)
Rural (< 10,000)	0.08 (0.07 – 0.10)
Time of call (Ref: 18:00–23:59)	
00:00‒05:59	0.68 (0.17 – 2.70)
06:00‒11:59	1.09 (0.13 – 9.43)
12:00‒17:59	1.16 (0.12 – 11.6)
Weekday (Ref: Wednesday)	
Sunday	0.93 (0.15 – 4.67)
Monday	0.78 (0.16 – 3.73)
Tuesday	1.07 (0.13 – 9.13)
Thursday	1.82 (0.04 – 67.7)
Friday	0.89 (0.15 – 5.23)
Saturday	0.76 (0.17 – 3.40)
Season (Ref: Spring (Mar 20– Jun 20))	
Summer (Jun 21‒Sep 22)	1.25 (0.10 – 15.2)
Fall (Sep 23‒Dec 20)	0.96 (0.14 – 6.49)
Winter (Dec 21‒Mar 19)	0.85 (0.15 – 4.67)
COVID-19 restrictions^‡^ (Ref: No)	
Yes	0.013 (0.013 – 0.014)***
Type of call (Ref: Supervised consumption)	
Mental health only	1.03 (0.12 – 8.59)
Info and resources	0.08 (0.07 – 0.10)*
Other/unspecified calls	0.59 (0.16 – 2.11)
Substances used (Ref: Opioids)	
Cocaine	0.61 (0.18 – 2.08)
Methamphetamines	0.38 (0.17 – 0.85)
Depressants^†^	0.12 (0.09 – 0.15)
Cannabis	0.17 (0.12 – 0.25)
Other substances	0.11 (0.08 – 0.14)
Unknown substances	1.21 (0.10 – 14.29)
Opioids and methamphetamines	0.46 (0.18 – 1.19)
Opioids and depressants^†^	0.16 (0.11 – 0.22)
Cocaine and methamphetamine	0.23 (0.14 – 0.38)
Polysubstance	0.55 (0.18 – 1.66)
No substance	0.83 (0.15 – 4.45)
Route (Ref: Injection)	
Smoking	1.16 (0.11 – 11.9)
Oral	0.96 (0.14 – 6.50)
Other routes	1.91 (0.04 – 94.8)
Unknown routes	0.84 (0.15 – 4.71)
No route	1.63 (0.06 – 42.1)
Polyroute	1.73 (0.06 – 52.7)
Insufflation/snorting	0.46 (0.17 – 1.16)
Peer support/active listening (Ref: No)	
Yes	1.63 (0.06 – 40.8)*
Previous required EMS dispatch on the NORS line (Ref: No)	
Yes	0.11 (0.08 – 0.13)
Previous overdose event on NORS line (Ref: No)	
Yes	2.29 (0.02 – 222.2)
Previous mental health adverse event on NORS line (Ref: No)	
Yes	0.25 (0.14 – 0.45)

*p*-value: * < 0.05, ** < 0.01, *** < 0.001

^**‡**^For this project, we define the duration of the Canadian COVID-19 restrictions as between March 1, 2020, and March 1, 2022

^**†**^Include depressants such as benzodiazepines and alcohol

## Footnotes

1 This was done in cases such as prank calls or emergencies wherein standard demographic information could not be collected.

## Code availability

Available from the corresponding author upon reasonable request.
